# Guidelines and metadata model for a repository of volcanic samples

**DOI:** 10.1007/s00445-025-01816-1

**Published:** 2025-04-09

**Authors:** Rosa Anna Corsaro, Claudia D’Oriano, Andrea Di Muro, Adelina Geyer, Lucia Gurioli, Lucia Pappalardo, Maddalena Pennisi, Massimo Pompilio, Claudia Principe, Giuseppe Re

**Affiliations:** 1 Istituto Nazionale di Geofisica e Vulcanologia, Sezione di Catania, Osservatorio Etneo, Catania, Italy; 2 Istituto Nazionale di Geofisica e Vulcanologia, Sezione di Pisa, Pisa, Italy; 3 CNRS, UMR 5276, LGL–TPE, Université Lyon 1, OSUL, Villeurbanne, France; 4 Geociències Barcelona (GEO3BCN), CSIC, Lluís Solé i Sabarís s/n, 08028 Barcelona, Spain; 5 Laboratoire Magmas et Volcans, Université Clermont Auvergne, CNRS, IRD, OPGC, Clermont-Ferrand, France; 6 Istituto Nazionale di Geofisica e Vulcanologia, Sezione di Napoli, Osservatorio Vesuviano, Naples, Italy; 7 Istituto di Geoscienze e Georisorse, Consiglio Nazionale Delle Ricerche, Pisa, Italy

**Keywords:** Rock repository, Volcanic samples, Metadata model, International database, Open science

## Abstract

The volcanological community manages heterogeneous types of data acquired during research projects and monitoring activities of volcanoes. These data consist of both continuous and discrete measurements and observations, which are carried out by ground-based networks and remote sensing instruments, or during field surveys and laboratory analyses. Many types of volcanological research are largely based on the accurate sampling of rocks erupted during past and ongoing volcanic activity. Each sample represents a “physical object” which should be identified and archived before part of it is removed for analytical purposes. In this context, we recommend assigning the collected samples unique and persistent identifiers, such as the International Generic Sample Number (IGSN). However, although the IGSN allows recording the most essential information of the collected samples (e.g. geographic location, sampling method, and collector), the predefined metadata fields are not exhaustive for volcanic samples, which require additional information such as type and timing of the eruptive event, sample temperature, and texture. Here we design the guidelines necessary to facilitate communication between and search of multiple sample repositories and databases run by disparate institutions. To this aim, we build a metadata model, which integrate the IGSN metadata with supplementary information necessary for the monitoring and research activities carried out by the volcanological community. The long-term curation of collected materials is an important investment for the future. Indeed, these collections are a resource for the production of volcanological data, they reduce the need for repeated sampling, they preserve samples that can no longer be collected, and they allow repeat analyses to be made. The primary aim of this work, based on discussion within the EUROVOLC project, is to provide the basic information for populating a relational database structure in the future for the description of different volcanic samples, physically located in different physical repositories and institutions, in order to facilitate future sharing between different groups of scientists and more complete volcanological studies, by means of the proposed metadata structure.

## Introduction

Rock and sediment sampling for physical, chemical, and mineralogical analysis is a fundamental aspect of volcanological research. Depending on the specific context, sampling is carried out with different methods and instruments (Gurioli et al. 2015). Still active or solidified lava flows require a core bar or a hammer (Harris et al. 2017), while pyroclastic material of ongoing eruptions or stratigraphic sequences can be sampled by hand picking and/or by using a set of dedicated tools (e.g. plastic sheets, buckets, core drilling, shovel, trowel, filters, and disdrometers; Re et al. 2021, 2022). Volcanic samples from dredges are generally wrenched with the winch (Barker et al. 2012; Berthod et al. 2022), while core samples from boreholes are collected with different techniques (e.g. diamond, air core, percussion, and continuous or destruction drilling; Nakagawa et al. 2012; Di Roberto et al. 2021; Macrì et al. 2023). Regardless of their origin, volcanic samples usually represent the unique “mementos” of past eruptions, since lava flows and pyroclastic deposits are progressively eroded by atmospheric agents, covered by subsequent eruptions, or reduced/remobilized due to anthropogenic actions. Furthermore, the advancement of new analytical techniques has greatly increased the amount and the variety of samples, as well as the data produced in laboratories, whose management requires a proper structure for an effective sharing and utilisation by a wide audience, adhering to the principles of FAIR (Findability, Accessibility, Interoperability, and Reuse). In response to this need, numerous multi-level databases, providing interactive exploration by users, have been developed. Most of them are focused on archiving data resource, such as geochemical rock analyses in GEOROC (http://georoc.mpch-mainz.gwdg.de/georoc/) or in PetDB of EarthChem (http://www.earthchem.org/); others are physical sample repositories or collections such PWD (Petrology and Workspace Database, https://petro.wovodat.org/#/home) and, specifically for volcanic rocks, DYNVOLC (OPGC centre de données-DYNVOLC), AntTephra (https://www.tephrochronology.org/AntT/about.html), Tephrabase (https://www.tephrabase.org/background.html), RESET (https://c14.arch.ox.ac.uk/reset/), Polar Rock Repository (https://prr.osu.edu/), MNA (https://mna.it/collezioni/catalogo-rocce-sede-di-siena), and VolcAsh DB (https://volcashdb.ipgp.fr/; Benet et al. 2024).

In recent decades, the establishment of European Research Infrastructures, such as the European Plate Observing System (EPOS, https://www.epos-eu.org), has played a crucial role in providing Data, Data products, Software and Services (DDSS) designed for the European scientific community. Preceding and synchronously with EPOS, other European projects, such as FUTUREVOLC (2012–2016) and MEDSUV (2013–2016) focused on volcanology and implemented the e-infrastructures for European Volcano Supersites. Subsequently, EUROVOLC (2018–2021), promoted integration within the European volcanological community. Notably, access to national and regional research infrastructures has been promoted by Trans-National Access (TNA), which funded calls for European researchers, in projects like EUROVOLC (https://project.eurovolc.eu/) and Geo-Inquire (https://www.geo-inquire.eu/).

Within this framework, the conception of an international open-access and widely accepted database for volcanic rock samples emerges as a critical necessity. This database requires a structure for documenting where samples are located along with their associated metadata and data, thereby enhancing the value and facilitating future use of the samples. Moreover, it is imperative for the database to be interconnected across various institutions, streamlining the process of archiving, accessing, and sharing samples. This is not merely a matter of convenience, rather it constitutes an essential step towards facilitating the exchange of samples among rock repositories of different institutions and permitting collaborative research.

The metadata structure of each repository must be conceived in order to facilitate the workflow: planning, sampling, storage and preservation of samples, processing analysis, and data sharing in order to facilitate interoperability with other disciplines (e.g. seismology, geophysics, fluid geochemistry; Andrews et al. 2022). To this end, we present the work conducted by several European research institutions of Italy, France, and Spain as part of the EUROVOLC Project. Specifically, our work focuses on structuring the guidelines for metadata associated with the collections of volcanic rock samples and takes steps towards implementing access to collections of magmatic rocks, according to the principles inspiring “open science”.

Our efforts fit closely into a broader context where the definition and organisation of metadata in well-structured and shared databases represent an immediate challenge that the volcanological community must face. Specifically, we aim to shape and expand the data associated with samples of rock repositories to satisfy the needs of the entire volcanological community, encompassing samples acquired during ongoing eruptive crises as well as in other geological contexts such as historical eruptions, paleoclimatic (ice, marine, lacustrine, caves) records, oceanographic campaigns, geoarcheological excavations etc. In this study, we refer as “repository” to the data storage system, and we use “physical repository” for the place where physical samples are stored.

## The sample’s label: the choice of IGSN identifier

Scientific papers and databases generally handle data derived from samples, which are usually named or labelled based on non-standardised criteria. It is therefore not uncommon that names of sample cause some misunderstanding; they often follow a structure based on toponyms or location acronyms, resulting in different studies of different samples using identical sample numbers or names. In addition, another recurrent issue is that samples are sometimes renamed during the course of a study, leading to multiple labels identifying the same object. For this reason, the implementation and utilisation of an international identification system for samples have become increasingly essential.

The International Generic Sample Number (IGSN), provided by SESAR (System for Earth Sample Registration), more recently by SESAR2 (https://www.geosamples.org/) and other allocating agents, is a progressive alphanumeric code that uniquely and globally identifies samples taken from natural environments, together with related sampling features (https://ev.igsn.org/). In Europe, the adoption of IGSN is steadily increasing among institutions managing collections of physical samples. Following this trend, in countries such as France the use of IGSN to identify samples from offshore campaigns is already mandatory. It is evident that the geoscience community derives significant benefits from the use of IGSN. Firstly, a unique identifier for a sample allows the integration of data produced in different labs or published in different articles or databases. Additionally, it improves the interoperability of distinct databases, facilitating the sharing and integration of multidisciplinary data. Moreover, it enhances the efficiency of sample utilisation by making them accessible to scientists through the Global Sample Catalog, where IGSN metadata are available.

While IGSN metadata satisfies the requirements for sample identification and geolocation (Andrews et al. 2022), for a collection of volcanic rocks, it lacks the comprehensive details to fully describe the diverse context of sampling of volcanic rocks, which encompass samples collected during an ongoing (syn-eruptive) eruption to those of historical activity retrieved from outcrops, stratigraphic sections, dredges, or boreholes. Hence, when designing the present guidelines to metadata structure for a repository of volcanic rock samples, we incorporate most of the metadata requested by SESAR while integrating additional information tailored to the specific requirements of the volcanological community.

## Structure of volcanic rocks metadata

The present state of most rock physical repositories, especially those housing older sample collections, typically involves one or more rooms of various sizes, furnished with shelves of boxes containing samples. Each box is labelled, as well as the samples contained within boxes and/or shelves. Generally, there is a list associated with each box, either digitised or, in some cases, still on paper, detailing its content. The information on the samples can be more or less accurate, but, especially for older samples, it rarely goes beyond an acronym, collection year, name of the sampler, and a brief description of the sampling location; complementary information often only lies in the hands of the sampler. To overcome this rather imprecise condition, first of all, the volcanological community needs a detailed and shared outline of the information that must accompany a sample to make it usable and shareable. This set of information composes the metadata to be organised in a Relational Database structure in the near future. The main concepts behind a relational database, well known in literature, are presented in Appendix Fig. 2.

**Fig. 1:**
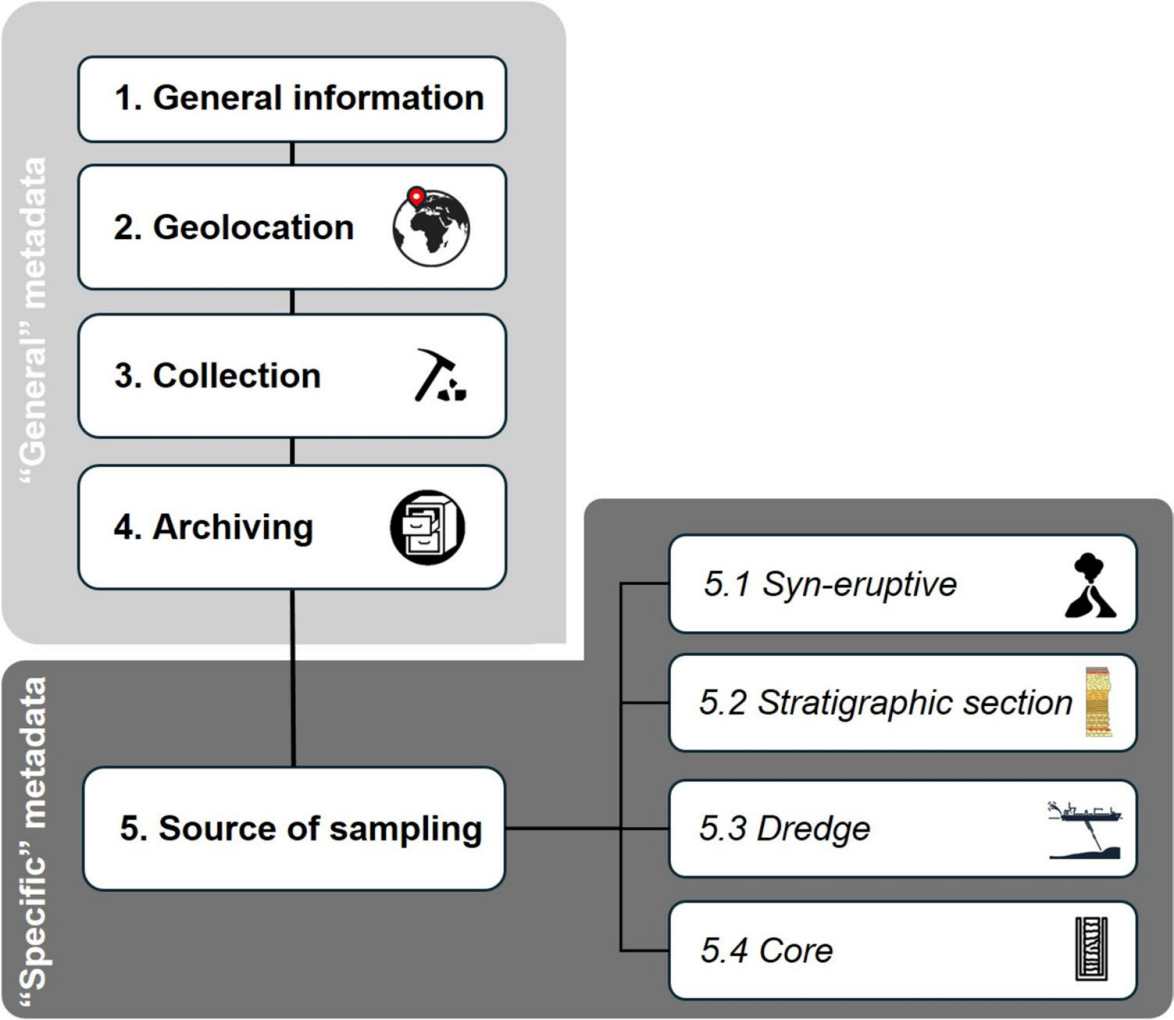
Metadata structure proposed for a repository of volcanic samples

Here we present the guidelines for a metadata structure organised as illustrated in Fig. 1, comprising two types of descriptors. Categories and related vocabularies must be machine-readable, consistent and interoperable with those of the largest geochemical (EarthChem/PetDB, DIGIS/Georoc, EarthRef/GERM, Astromat,) and mineralogical (MinDat) databases.

The “General” metadata (boxes 1 to 4 in Fig. 1) include the basic information of a sample, while the “Specific” metadata (box 5 in Fig. 1) provide additional details essential to define the volcanic context related to sampling strategies (boxes 5.1 to 5.4 in Fig. 1).

### “General” metadata

This section includes all the information which are essential to characterise a generic sample. In particular:

1. General information: this comprises basic information aimed at identifying the sample and providing an initial characterisation, including classification, description of the sample texture and physical characteristics visible to the naked-eye or with low magnification lenses, as well as geological unit, age, and eruption. It is also desiderable to include the reference (preferably using DOIs, if available) of the scientific papers or research works (e.g. PhD Theses) that investigate the sample in order to enlarge background knowledge. The terminology of the field “Sample description” follows the hierarchical classification of the BGS (Gillespie and Styles 1999) partly based on the IUGS recommendations (Le Maitre et al. 2002), for igneous rock types (https://www.bgs.ac.uk/technologies/bgs-rock-classification-scheme). This choice must facilitate user accessibility by allowing easy integration in reference catalogues (e.g. SESAR) or data models (e.g. ODM2, https://www.odm2.org/).
2. Geolocation: this reports information concerning the location (latitude, longitude, and elevation) of the sample and of the physiographic feature (e.g. a volcano or a volcanic area) from which the sample comes from.
3. Collection: this comprises information illustrating the sample’s collection in terms of institution/project that funds the activity, information on cruise/dredge, personnel that perform the sampling, date, timing, and methods of sampling, as well as weight, alteration, photo of the collected material.
4. Archiving: this collects the basic information concerning the Institution and scientist in charge of the rock physical repository, as well as the details of sample’s position (box, shelf) and comprises, if present, any type of ready-to-use preparation, including daughter samples.

### “Specific” metadata

This section encompasses all the volcanological facets associated with various sources/conditions of sampling and methodology, offering a deeper understanding of the volcanic context. Gathering such information significantly enhances the value of the sample, providing vital details for subsequent study and interpretations.

#### Syn-eruptive sampling

Samples collected during an eruption, or soon thereafter, primarily consist of lava flow fragments and pyroclasts. Their study is crucial particularly for volcano observatories and Institutions in charge of the volcanological/petrological monitoring (Re et al. 2021). Indeed, syn-eruptive samples provide information for evaluating the temporal evolution of the magmatic feeding system, as well as the dynamics and the intensity of the eruption (Watanabe et al. 1999; Cashman and Hoblitt 2004; Corsaro et al. 2009; Di Muro et al. 2014; Gurioli et al. 2018; Vlastélic et al. 2018; Benet et al. 2021; Thivet et al. 2021; Corsaro and Miraglia 2022; D’Oriano et al. 2022; Andronico et al. 2024), allowing derivation of eruption parameters, such as magma volume, total grain size distribution, or column height (Biass and Bonadonna 2011 and references therein).

Samples should be associated with information about: (1) the description of the activity (e.g. effusive, spattering, Strombolian explosions, paroxysm, and ash emission); (2) the start and end dates of the eruption; (3) the description of the eruption site (e.g. name of the crater, altitude of a sector of the eruptive fissure, coordinates, other); (4) the geographical coordinates and elevation of the eruption site, above or below for underwater sample sets.

For samples collected during or soon after an eruption, specific information includes: (1) the eruptive temperature (in case of lava flows); (2) the conditions of transport medium advancement; and (3) the position of the sample within a lava flow (at vent, front, etc.). For pyroclasts (ash, lapilli, and bombs/blocks), additional essential information includes (1) the extent of the sampled area; (2) the thickness of the deposit or lava flow unit; and (3) the weight of the collected pyroclastic sample by unit area.

#### Stratigraphic section

A stratigraphic section is a vertical sequence formed by the accumulation of volcanic products, including both lava flows and pyroclastic rocks. It provides the temporal succession of eruptive events, occurring at a specific distance from the volcanic source and within a particular environment (subaerial, lacustrine, submarine, englacial sequence, etc.). An old lava flow, which is not a syn-eruptive product, can be considered as a unique layer of a stratigraphic section.

A comprehensive description of a stratigraphic section includes both the general information of the section and the details on the sampled layers (Thivet et al. 2020; Di Muro et al. 2021; Prival et al. 2022; Lacombe et al. 2024; Re et al. 2025; Shajahan et al. 2024). The former includes information about the name of the section, its orientation, and thickness. Additionally, a measured stratigraphic log should always be associated, or a graphical representation of the section, either as a photo or as a schematic, highlighting the position of the sample collected within the section. Other information pertains to the layers to which the sample belongs to (1) the geometry (thickness, width, length, slope, orientation); (2) the type of contact between the sampled layer and the top and bottom layers (e.g. conformity, disconformity, angular unconformity, non-conformity, intrusive, fault/tectonic, and presence of palaeosols); (3) the type of lateral continuity (e.g. pinch-out, intertonguing, lateral gradation, continuous); and (4) the description of internal primary structure. For pyroclasts, it is important to indicate the grain size and componentry of the deposits, the degree of grading, sorting, and the sedimentary structures (lenses, plane, or cross stratifications). For lava samples, it is relevant to add information about the lithology (composition and texture), the structures (beddings, shearings, breccia, columns, substructures), and the movement patterns (ramps, deformations, stretching, scritching, cooling/quenching).

The details of the sampled layers aim to recover information on (1) freshness (colour), (2) genesis (e.g. lava flow, pyroclastic rocks, spattering), (3) mechanisms of deposition (e.g. fallout or tractional), and (4) the degree of the magma fragmentation (grain size distribution) and lava flow type.

Eventually, the identification of underlying soils, or any other marker layers, associated with the eruption deposit, can aid in the spatial and temporal correlation of the layers sampled at different locations.

#### Dredge

Despite the development of Remotely Operated Vehicle and human-operated submersibles (e.g. Nautile, Hekinian et al. 2000; Jason, Murch et al. 2019), dredging operations remain fundamental and are the more practical way for sampling and characterising submarine volcanic structures and related products. This sampling method allows collecting a large quantity of material (up to 1 t) statistically representative of the studied area.

In recent decades, more accurate navigation systems and improved geophysical survey methods have allowed researchers to monitor the dredge operation (in time and space) and to ensure that the dredge sampling is representative of the investigated geological terrains. The most recent protocol used for dredging during the on-going volcano-tectonic crises of Mayotte is reported in Berthod et al. (2022). The description of dredge samples includes: (1) general information about the cruise and the ship and specific details about (2) the topographic profile along which the dredge is deployed, (3) the release of the winch, (4) a preliminary lithological and textural description of the collected material, and (5) photos of the bulk products (both within the dredge and with the rocks on the deck).

#### Core

Boreholes are drilled into the Earth’s crust, penetrating the sedimentary cover to extract core samples, allowing the investigation of layers up to hundreds of meters below the surface. In recent years, cored samples have emerged as indispensable tools for tephrochronology, as well as the study of distal tephra layers within continental and marine sedimentary and glacial sequences (Dunbar and Kurbatov 2011; Narcisi et al. 2005, 2012; Lowe et al. 2015; Giaccio et al. 2017; Smith et al. 2019; Di Roberto et al. 2023). This discipline extends beyond volcanology, finding applications in diverse fields such as tectonics, paleoenvironment, paleoclimatology, and glaciology (Del Carlo et al. 2009; Paulsen et al. 2012; Wilson et al. 2012, Di Roberto et al. 2021).

The description of core samples encompasses a set of information, such as the (1) borehole name and location, (2) the diameter and total thickness, and (3) the starting and ending depth. Successively, it includes information about the core, such as (4) the quality of the recovery and (5) the position of the sample along the borehole. Finally, information about the stratum to which the sample belongs is also provided, such as (6) the position along the borehole, the thickness, and the lithological and textural features. The schematic borehole log and photographs can complete the documentation.

## Description of volcanic rocks metadata

The full description of data and metadata suggested is provided in Table 1. The different columns indicate the NAME (the name of the field), DESCRIPTION (the content of the field), OPTIONALITY (if the information is mandatory or optional), EXAMPLE (an example to clarify the “description”), and FORMAT (the format of data), VOCABULARY (terms to use, taken from a glossary, see the footnote of Table 1). The fields that overlap with the IGSN archive are written in bold in the column “Name” (Table 1). This table is not exhaustive, but it represents a starting point to gather the volcanology community around the need to find a common language and standardisation to organise the different sample collections.

**Table 1:** Description of data and metadata of volcanic samples of a rock repository. In the column “Name”, the fields that overlap with the IGSN archive are written in bold; in the column “Vocabulary”, the capital letters refer to the URL reported in the footnote

Name	Description	Optionality	Example	Vocabulary*	Format
**1.** **GENERAL INFORMATION**					
**Sample name**	Name of the sample	M	RN1-XB		Text
**IGSN**	International Generic Sample Number (IGSN) that uniquely identifies the sample. It is assigned by SESAR (System for Earth SAmple Registration) or any other allocating agent	O	10.58052/IEGVF21RN1XB		Text
**Release date**	Date when the IGSN is released	O	2023–02–18		Date YYYY-MM-DD
**Material**	Type of material	M	Rock, Sediment, Tephra, Other	A	Text
**Sample type**	Type of sample and related sampling features collected from the natural environment	M	Individual sample, core, cuttings, dredge, other	B	Text
**Classification (rock)**	Formal rock categorization of sample	M	Igneous > volcanic, sedimentary, xenolithic, unknown, other	C	Text
**Sample description**	Terminology that identifies the physical characteristics of the sample visible in the field to naked-eye or with low magnification lenses	M	Lava flow, pyroclastic flow, pyroclastic fall etc	D	Text
Texture	Description of the texture of the sample	O	(e.g. massive, vesicular, laminar, scoriaceous, pumiceous, and obsidian-like)		Text
Volcano name	Name of the volcano according to the GVP (Global Volcanism Program), Smithsonian Institution	M	Stromboli	E	Text
Volcano ID	The volcano number that uniquely identifies it according to the GVP (Global Volcanism Program), Smithsonian Institution	M	211,040	E	Text
Eruption ID	The eruption number that uniquely identifies it according to the GVP (Global Volcanism Program), Smithsonian Institution	O	2024 CE	E	Text
**Geological unit**	Lithostratigraphic unit to which the sample belongs, according to scientific literature data. Lithostratigraphic unit is a body of rock that is defined on the basis of its lithologic properties and stratigraphic relations. It is the basic units of geologic mapping (from IUGS-ICS: International Commission on Stratigraphy)	O	Serra delle Concazze		Text
	Epoch of the sample according to IUGS-ICS: International Commission on Stratigraphy)	M	Holocene		Text
Reference(s)	Reference(s) of scientific paper(s) that provides details/results achieved on the studied sample	O			Text
Notes	A free text for any information to add to general information	O			Text
**2. GEOLOCATION**					
**Sample** **latitude**	Latitude of the sample	M	42.142167		Number DD.DDDDDDD WGS 84, decimal degrees. Lat. are − 90 to 90 (0 = equator; − numbers are South, + numbers North)
**Sample longitude**	Longitude of the sample	M	− 2.554555		Number DDD.DDDDDD WGS 84, decimal degrees. Long. are − 180 to 180 (0 = Greenwich), − numbers are West, + numbers are East)
**Sample elevation**	Elevation of the sample	O	1850 m asl		Number NNNN.N m asl (above sea level) or bsl (below sea level)
**Navigation type**	Navigation type used to determine latitude and longitude	O	GPS	F	Text
**Primary physiographic feature**	A description of the landscape and landforms	M	Volcano	G	Text
**Name of physiographic feature**	Physiographic feature according to the names of the GNS (Geographic Names Server)	M	Stromboli	H	Text
**Country**	Country where the physiographic feature is located according to the names of the GNS (Geographic Names Server)	M	Italy	H	Text
**State/province**	State/region/province where the physiographic feature is located according to the names of the GNS (Geographic Names Server)	M	Sicily	H	
**Locality description**	Locality where the physiographic feature is located according to the names of the GNS (Geographic Names Server)	O	n/a	H	Text
**Latitude**	Latitude of the physiographic feature (in case of an area it is the starting latitude)	M	42.142167		Number DD.DDDDDDD WGS 84, decimal degrees. Lat. are − 90 to 90 (0 = equator; − numbers are South, + numbers North)
**Latitude (end)**	In case of an area, it is the ending latitude of the physiographic feature	O	42.142290		Number DD.DDDDDDD WGS 84, decimal degrees. Lat.are − 90 to 90 (0 = equator; − numbers are South, + numbers North)
**Longitude**	Longitude of the physiographic feature (in case of an area it is the starting longitude)	M	− 2.554555		Number DDD.DDDDDD WGS 84, decimal degrees. Long. are − 180 to 180 (0 = Greenwich, − numbers are West, + numbers are East)
**Longitude (end)**	In case of an area, it is the ending longitude of the physiographic feature	O	− 2.544350		Number DDD.DDDDDD WGS 84, decimal degrees. Long. are − 180 to 180 (0 = Greenwich, − numbers are West, + numbers are East)
Notes	A free text for any information useful to describe the geolocation	O			Text
**3. COLLECTION**					
**Field program/cruise**	Name of the program, cruise, or project funding the activity of sampling	M	CRU-1		Text
Institution	Name of Institution leading the activity of sampling	M	University of Barcelona		Text
Company contractor	Contractor company name	O	Best Company spa		Text
Cruise locality	Locality where the cruise takes place according to the names of the GNS (Geographic Names Server)	O	Mediterranean Sea	H	Text
Ship name	Name of the ship	M	Big Vessel		Text
Leg name	Name of the LEG, which is a distinct part of the overall cruise	M	LEG-1		Text
Scientist LEG	Last and first name of scientist in charge of the LEG	M	Newton John		Text
Cruise departure locality	Locality where the cruise starts according to the names of the GNS (Geographic Names Server)	M	Genova, Italy	H	Text
Cruise departure date	Cruise starting date	M	2023–03–10		Date YYYY-MM-DD
Cruise arrival locality	Locality where the cruise ends according to the names of the GNS (Geographic Names Server)	M	Genova	H	Text
Cruise arrival date	Cruise ending date	M	2023–03–25		Date YYYY-MM-DD
**Collector/chief scientist**	Last and first name of the sample’s collector	M	Rossi Francesco Giuseppe		Text
**Collector/chief scientist Address**	Email of the sample’s collector	M	Rossi@gmail.rst.com		Text
**Collection date**	Date of sample collection (in case of a time frame, it is the starting date of sample collection)	O	2020–09–18		Date YYYY-MM-DD
**Collection date (end)**	In case of a time frame, it is the ending date of sample collection	O	2020–09–19		Date YYYY-MM-DD
Collection date precision	Valuation of the precision of the sample’s collection date		(e.g. exact, unknown, presumed)		Text
**Collection time**	Time of sample collection (in case of a time frame, it is the starting time of sample collection)	O	10:05:00		Time hh:mm:ss [hh] refers to an hour between 00 and 24; [mm] refers to a minute between 00 and 59.[ss] refers to a second between 00 and 60
**Collection time (end)**	In case of a time frame, it is the ending time of sample collection	O	10:07:00		Time hh:mm:ss [hh] refers to an hour between 00 and 24; [mm] refers to a minute between 00 and 59.[ss] refers to a second between 00 and 60
**Collection method**	Method by which a sample has been collected	M	Manual > Hammer	I	Text
Collection method description	Description of the sampling method	O	(e.g. total mass, hand picking, water quenching)		Text
**Weight**	Weight of the sample	O	1450.00 g		Number NNNN.NN g (gram)
Alteration	Description of the state of alteration of the sample	O	(e.g. changes in colour, presence of secondary minerals,)		Text
Photo	Photo of the collected sample	O			Image (.jpg;.tiff)
Photo description	A free text for any information useful to describe photo of the collected sample	O			Text
Notes	A free text for any information useful to describe the collection	O			Text
**4. ARCHIVING**					
**Current archive**	Locality of the rock repository according to the names of the GNS (Geographic Names Server)	M	Pisa	H	Text
**Current archive contact**	Last and first name of the contact person of the rock repository	M	Smith Jane		Text
Preparation	Sample preparation before storage	O	(e.g. drying, sieving, cutting)		Text
Racking	Identifier of the raking where the sample is archived in the rock repository	O	A		Text
Shelf	Identifier of the shelf where the sample is archived in the rock repository	O	A10		Text
Box	Number of the box containing the sample	O	24		Text
Notes	A free text for any information useful to describe the archiving	O			Text
**5.1** **SYN-ERUPTIVE**					
Sample eruption date	Date when the sample was erupted	O	2020–12–13		Date YYYY-MM-DD
Eruption date precision	Valuation of the precision of the sample’s eruption date	O	(e.g. exact, unknown, presumed)		Text
Sample eruption time	Time when the sample was erupted (GMT)	O	10:07:00		Time hh:mm:ss [hh] refers to an hour between 00 and 24; [mm] refers to a minute between 00 and 59.[ss] refers to a second between 00 and 60
Eruption start	Start date of eruption	O	2020–12-01		Date YYYY-MM-DD
Eruption end	End date of eruption	O	2021–01–24		Date YYYY-MM-DD
Type of activity	Description of the type of activity of the eruption	M	(e.g. effusive, spattering, paroxysm, Strombolian explosions, ash emission)		Text
Eruption site	Description of the eruption location useful to identify the eruptive vent/fissure producing the sample	O	(e.g. name of the crater, altitude of a sector of eruptive fissure)		Text
Latitude	Latitude of the eruption site (vent/eruptive fissure)	M	42.142167		Number DD.DDDDDDD WGS 84, decimal degrees. Lat. are − 90 to 90 (0 = equator; − numbers are South, + numbers North)
Latitude (end)	In case of an area/fissure, it is the ending latitude of the eruption site	O	42.142169		Number DD.DDDDDDD WGS 84, decimal degrees. Lat. are − 90 to 90 (0 = equator; − numbers are South, + numbers North)
Longitude	Longitude of the eruption site (vent/eruptive fissure)	M	− 2.554555		Number DDD.DDDDDD WGS 84, decimal degrees. Long. are − 180 to 180 (0 = Greenwich, − numbers are West, + numbers are East)
Longitude (end)	In case of an area/fissure, it is the ending longitude of the eruption site	O	− 2.554558		Number DD.DDDDDDD WGS 84, decimal degrees. Lat. are − 90 to 90 (0 = equator; − numbers are South, + numbers North)
Elevation	Elevation of the main eruption site (vent/eruptive fissure)	M	1200 m asl		Number NNNN.N m asl (meter above sea level) or bsl (below sea level)
Temperature	The measured temperature of a lava flow or deposit	O	1050.0 °C		Number NNNN.N °C
Temperature method	The method used for temperature estimation	O	(e.g. thermocouple, IR camera)		Text
Location	General statement describing where the sample is located	O	(e.g. vent/eruptive fissure, ephemeral vent, lava channel, lava front, levees, distal area, proximal area, intermediate area)		Text
Position	The portion of the lava flow and or the pyroclastic deposit which has been sampled	O	(e.g. surface, intermediate, base, inside lava tunnel)		Text
Advancement	Conditions of lava flow advancement during the sampling	O	(e.g. ongoing, stopped)		Text
Width area	Width of the sampled area	O	25.0 cm		Number NN.N cm (centimeter)
Length area	Length of the sampled area	O	20.0 cm		Number NN.N cm (centimeter)
Thickness area	Thickness of the sampled area	O	2.0 cm		Number NN.N cm (centimeter)
Weight/area	Ratio between the weight of a sample and the area of its deposition	O	2.9 g/cm^2^		Number NN.N g/cm^2^ (gram/centimeter^2^)
Notes	A free text for any information useful to describe the syn-eruptive sample	O			Text
**5.2** **STRATIGRAPHIC SECTION**					
Section name	Name of the log (section)	O	Log 127		
Section orientation	Orientation of the face of the section	O	NNE-SSW		Text (max 50 character)
Section thickness	Thickness of the whole outcrop/log	M	4 m		Number NNN.NN m (meter)
Layer strike	Strike of the sampled layer: intersection of an inclined plane with a horizontal plane expressed as azimuth in the horizontal plane (a horizontal angle measured clockwise from true north, from 000 to 360°)	O	225°		Angle 000°
Layer inclination	Inclination of the sampled layer: the vertical angle, measured downward, between the horizontal and the line of greatest slope in an inclined plane. The dip direction is always 90° clockwise from the strike direction	O	64°		Angle 00°
Layer position	Position of the sampled layer along the section. It is expressed as the distance from the top of the layer to the top of the section	M	5 m		Number NNN.NN m (meter)
Layer thickness	Thickness of the sampled layer	O	0.70 m		Number NNN.NN m (meter)
Sample vertical position	Position of sample along the section. It is expressed as the distance from the sample to the top of the layer to which it belongs	M	0.65 m		Number: NNN.NN m (meter)
Layer contact_top	Type of contact of the sampled layer with the top	O	(e.g. conformity, disconformity, angular unconformity, non-conformity, intrusive, fault/tectonic)		Text
Layer contact_bottom	Type of contact of the sampled layer with the bottom	O	(e.g. conformity, disconformity, angular unconformity, non-conformity, intrusive, fault/tectonic)		Text
Layer continuity	Type of lateral continuity of the sampled layer	O	(e.g. pinch-out, intertonguing, lateral gradation, continuous at section scale)		Text
Layer primary structure	Description of the internal primary structure of the sampled layer	O	(e.g. for pyroclastic layer: plane-parallel-bedding, cross-bedding, lamination, massive, vitrophyro) (e.g. for lava layer: beddings, shearings, breccia, columns, fractures, ramps, deformations, stretching, scritching, cooling/quenching, folds, foliation)		Text
Layer primary texture (lava)	Description of the internal constituents for lava	O	(e.g. vesicles: size, distribution, number/percentage, shape and orientation) (e.g. crystals (size, distribution, number/percentage, shape and orientation) (e.g. voids (size, distribution, number/percentage, shape and orientation) (e.g. enclaves (size, distribution, number/percentage, shape and orientation) (e.g. Fiamme (size, distribution, number/percentage, shape, orientation, gradient)		Text
Layer primary texture (pyroclastic deposit)	Description of the internal constituents for pyroclastic deposit	O	(e.g. grain arrangement: clast-supported, matrix-supported) (e.g. grain size: coarse, medium, fine) (e.g. sorting: well-sorted, poorly sorted) (e.g. grading: normal, reverse, non-graded) (e.g. componentry: e.g. monodispersal vs polydispersal and juvenile vs non-juvenile (e.g. morphology: e.g. sphericity, roundness, flatness)		Text
Markers	Description of stratigraphic markers	O	(e.g. soils underlying the sampled layer, marker layer, dikes, large ballistic bombs scattered in the layer, tree moulds)		Text
Log/schematic/photo	Log/schematic/photo of the stratigraphic section	O			Image (.jpg;.tiff)
Notes	A free text for any information useful to describe the stratigraphic section	O			Text
**5.3** **DREDGE**					
Dredge name	Name of the dredge	M	DR25-A		Text
Dredge starting water	Starting water depth of the dredge	M	850 m bsl		Number NNNN.N m bsl (below sea level)
Dredge ending water	Ending water depth of the dredge	M	450 m bsl		Number NNNN.N m bsl (below sea level)
Dredge starting winch	Starting winch release	O	n/a		Number NNN.N kN (kiloNewton)
Dredge ending winch	Ending winch release	O	n/a		Number NNN.N kN (kiloNewton)
Material description	Lithological and textural description of the collected material	M	(e.g. heterolithologic material of angular volcanic, and sedimentary fragments)		Text
Photo	Photo of bulk material	O			Image (.jpg;.tiff)
Notes	A free text for any information useful to describe the dredge	O			Text
**5.4** **BOREHOLE**					
Borehole name	Name of the borehole	M	DR25-UR		Text
Borehole location	Detailed information on the location of the borehole	O	Contrada Acquachiara, Maletto		Text
Borehole starting depth	Elevation of the borehole head	M	1350 m asl		Number NNNN.N m asl (meter above sea level) or bsl (below sea level)
Borehole ending depth	Elevation of the borehole bottom	M	1250 m asl		Number NNNN.N m asl (meter above sea level) or bsl (below sea level)
Borehole total thickness	Total thickness of the drilled sequence	O	100 m		Number NNN.N m
Borehole diameter	Diameter of the borehole	O	10 cm		Number NN.N cm
Core recovery	The length of core recovered against the length drilled in a borehole	M	60%		Number NN %
Sample vertical position	Position of sample along the borehole. It is expressed as the distance from the sample to the borehole head	M	25 m		Number NNN.NN m (meter)
Stratum position	Position of the sampled stratum along the borehole. It is expressed as the distance from the top of the stratum to the borehole head	M	20 m		Number NNN.NN m (meter)
Stratum thickness	Thickness of the stratum to which the sample belongs		10 m		Number NNN.NN m (meter)
Stratum description	Lithological and textural description of the stratum to which the sample belongs	M	(e.g. dark-grey massive unaltered lava)		Text
Borehole drilling log	Vertical log of the drilling operation	M			Image (.jpg;.tiff)
Borehole lithol log	Vertical log of the drilled lithological sequence	M			Image (.jpg;.tiff)
Borehole sampling log	Vertical log of the sampling points	M			Image (.jpg;.tiff)
Notes	A free text for any information useful to describe the borehole	O			Text

*Terms which are not present in the list of vocabularies (URL) should be avoided, or eventually inserted under the voice “Other”

A) https://www.geosamples.org/vocabularies/material

B) https://www.geosamples.org/vocabularies/sample-type-object

C) https://www.geosamples.org/vocabularies/classification-rock

D) https://www.bgs.ac.uk/technologies/bgs-rock-classification-scheme/

E) https://volcano.si.edu/search_volcano.cfm

F) https://www.geosamples.org/vocabularies/navigation-type

G) https://www.geosamples.org/vocabularies/physiographic-feature

H) https://geonames.nga.mil/geonames/GNSHome/welcome.html

I) https://www.geosamples.org/vocabularies/collection-method

## Challenges for the future

The management of volcanic samples and their data poses numerous challenges for the volcanological community. Technological advancements continuously evolve metadata structures to accommodate emerging data types and measurement techniques. Furthermore, ensuring seamless integration and interoperability across diverse databases and platforms is crucial for international collaborations involving various institutions and technologies. To this end, standardising metadata across institutions and nations with different collection objectives, methods, equipment, and local practices, is a key goal.

The long-term curation of collected samples is an ongoing effort and requires sustained funding, allocation of human resources to preserve samples for future studies, and the availability of physical space for storing and curating samples. Managing historical collections, comprising samples dating back centuries preserved at Research Institutions or Museums (e.g. the collections of meteorites and the collections of samples carried out on exploration trips in the nineteenth century), poses an even greater challenge due to the lack of comprehensive information. Moreover, ethical considerations regarding sample collection from sensitive or culturally significant sites, necessitate a thoughtful approach to a conscientious sample usage.

Looking towards the future of the European volcanological community, our work represents the starting point for implementing criteria for the characterisation and classification of volcanic rock samples stored in physical repositories. The need to define and categorise samples at a European level goes beyond standardisation, as it is a strategic move towards harmonised volcanological practices useful for research and monitoring purposes. Furthermore, various disciplines studying volcanic products would benefit from a well-organised structure of volcanic rock repositories. As an example, accessibility to fresh volcanic samples from a rock physical repository could enhance future researches on the critical zone (i.e. the fragile skin of the Earth where rock meets life; Brantley et al. 2007), as soils and vegetation developed on volcanic rocks and pyroclastic products, with distinct timing and physico-chemical characteristics. In health studies, the characteristics of volcanic ash samples, such as clasts morphology and PM concentration, are fundamental to investigate toxicological properties (e.g. Horwell and Baxter 2006; Damby et al. 2017; Eychenne et al. 2022). Any global comparative study on acute and chronic exposures to volcanic emissions may need access to samples stored in a physical repository of volcanic solid products.

In the future, we envision the ability to query all rock repositories within EPOS. A prospective implementation of the data model discussed in this paper, foresees the development of a relational database structure permanently hosted by the Institutions participating in the EUROVOLC project, which can commit the necessary staff time and other resources to launch and maintain the database. The database structure will enable querying of all rock repositories via specific services, using a unified vocabulary and consistent criteria, in accordance with EPOS platform architectures. This collaborative approach is essential for ensuring the long-term sustainability of the infrastructure within EPOS.

This forward-looking initiative aims to streamline accessibility and research capabilities, fostering a harmonised system that will enhance collaboration within the volcanological community and implement services already provided by several European Institutions, such as the offer of volcanic samples stored in rock physical repositories for scientific study. In particular, within the EUROVOLC project, the rock repositories of Etna and Vesuvius Observatories of INGV, as well as the IGG-CNR Pisa Branch, actively participated in Trans-National Virtual Access Activities (TA/VA). These physical repositories provide access to carefully selected volcanic rock samples (e.g. 10.13127/ETNA/BKROCKCATALOG_1995_2012), following the initiation of calls for access and subsequent evaluation of submitted projects by a technical committee. The overarching goal of this project is to create opportunities for various stakeholders in volcanology, including research institutions, academics, students, and industries, to utilise facilities, data, and services, fostering cutting-edge research and implementing technological innovations.

## Appendix A

Figure 2

**Fig. 2:**
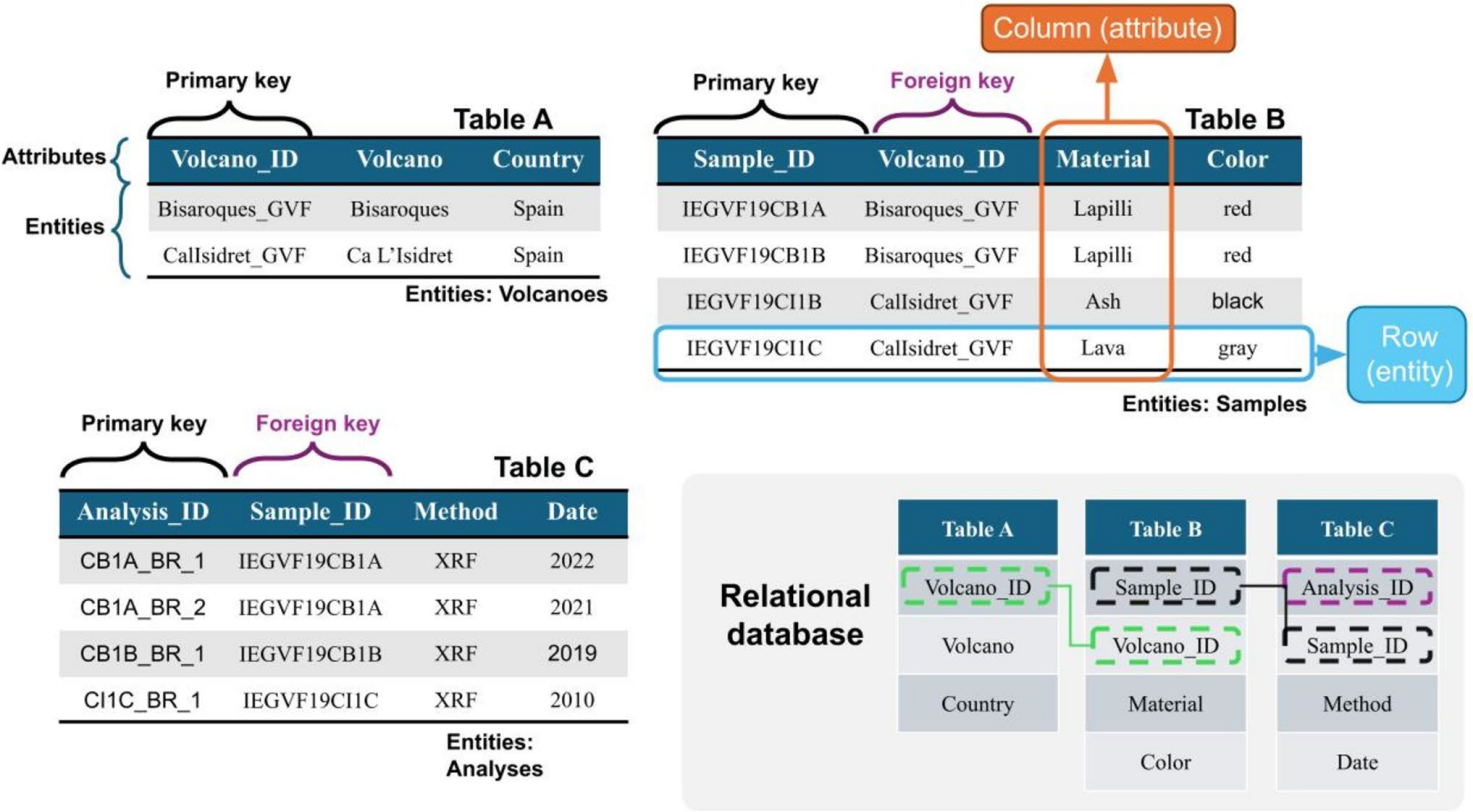
Schema of a relational database

## Data Availability

Metadata of the proposed model are not currently stored. If requested, Table 1 could be provided as an Excel file of Supplementary information.
